# Non-Thermal Technologies as Tools to Increase the Content of Health-Promoting Compounds in Whole Fruits and Vegetables While Retaining Quality Attributes

**DOI:** 10.3390/foods10122904

**Published:** 2021-11-23

**Authors:** Daniel A. Jacobo-Velázquez, Jorge Benavides

**Affiliations:** 1 Tecnologico de Monterrey, Escuela de Ingeniería y Ciencias, Av. General Ramón Corona 2514, Zapopan C.P. 45201, Jalisco, Mexico; 2 Tecnologico de Monterrey, Escuela de Ingeniería y Ciencias, Av. Eugenio Garza Sada 2501, Monterrey C.P. 64849, Nuevo Leon, Mexico; jorben@tec.mx

**Keywords:** controlled abiotic stresses, nutraceuticals, elicitation, functional foods, whole fruits and vegetables, stress-induced biosynthesis, secondary metabolites, innovative technologies, non-thermal technologies, health-promoting compounds

## Abstract

Fruits and vegetables contain health-promoting compounds. However, their natural concentration in the plant tissues is low and in most cases is not sufficient to exert the expected pharmacological effects. The application of wounding stress as a tool to increase the content of bioactive compounds in fruits and vegetables has been well characterized. Nevertheless, its industrial application presents different drawbacks. For instance, during the washing and sanitizing steps post-wounding, the primary wound signal (extracellular adenosine triphosphate) that elicits the stress-induced biosynthesis of secondary metabolites is partially removed from the tissue. Furthermore, detrimental reactions that affect the quality attributes of fresh produce are also activated by wounding. Therefore, there is a need to search for technologies that emulate the wound response in whole fruits and vegetables while retaining quality attributes. Herein, the application of non-thermal technologies (NTTs) such as high hydrostatic pressure, ultrasound, and pulsed electric fields are presented as tools for increasing the content of health-promoting compounds in whole fruits and vegetables by inducing a wound-like response. The industrial implementation and economic feasibility of using NTTs as abiotic elicitors is also discussed. Whole fruits and vegetables with enhanced levels of bioactive compounds obtained by NTT treatments could be commercialized as functional foods.

## 1. Introduction

Fruits and vegetables are essential sources of health-promoting compounds; thus, their consumption aids in the prevention of chronic and degenerative diseases [1]. However, the content of bioactive compounds in most fruits and vegetables is insufficient to exert the pharmacological effect expected of nutraceutical and functional foods [2]. Therefore, there is a need to find innovative technologies that increase the content of secondary metabolites with health-promoting properties in plant tissues. In this context, the application of controlled abiotic stresses during postharvest has been recognized as an effective strategy to elicit the secondary metabolism of fruits and vegetables, leading to the accumulation of bioactive ompounds [3,4]. Of the different postharvest abiotic stresses evaluated to induce the biosynthesis of plant bioactives, wounding stress has been recognized as one of the postharvest treatments with the highest impact on the activation of primary and secondary plant metabolism, generating significant increases in the accumulation of secondary metabolites [5,6,7]. However, the application of wounding stress at industrial scales presents different drawbacks. For instance, during the washing and sanitizing steps post-wounding, which are needed to ensure the safety of fresh-cut produce, the primary wound signal (extracellular adenosine triphosphate, ATP) that elicits the stress-induced biosynthesis of secondary metabolites is partially removed from the tissue [8]. Furthermore, detrimental oxidative reactions that affect the quality attributes of the fresh produce (i.e., color, flavor, and texture) are also activated by wounding [9]. Therefore, there is a need to search for technologies that emulate the wound response in whole fruits and vegetables while retaining quality attributes.

The application of non-thermal technologies (NTTs) as abiotic elicitors to induce the biosynthesis of bioactive compounds in plant foods was proposed by Jacobo-Velázquez et al. [10]. This proposal was based on previous reports where elicitation by NTTs was observed in different plant cell cultures [11,12,13]; thus, it was hypothesized that the results could be extrapolated to fruits and vegetables. In their initial proposal, Jacobo-Velázquez et al. [10] presented a hypothetical model indicating that NTTs could elicit the biosynthesis of secondary metabolites by inducing a wound-like response in plant tissues. In this context, it was stated that high hydrostatic pressure (HHP), ultrasound (US), and pulsed electric fields (PEF) generate cell membrane disruption by different driving forces [10]. The scientific community responded well to the concept of using NTTs as elicitors to induce the biosynthesis of bioactive compounds in plant foods, and several research groups have evaluated the potential of HHP, US, and PEF to induce the biosynthesis of secondary metabolites (i.e., carotenoids, phenolics, and glucosinolates) in different whole fruits and vegetables [14,15]. Furthermore, the hypothetical model proposed by Jacobo-Velázquez et al. [10] explaining the physiological mechanisms governing the biosynthesis of bioactive compounds induced by NTTs was recently revisited by López-Gámez et al. [14].

In the present article, the application of NTTs such as HHP, US, and PEF are presented as tools to increase the content of health-promoting compounds in whole fruits and vegetables by inducing a wound-like response. The main effects of NTTs on secondary metabolite biosynthesis and the quality attributes of whole fresh produce are discussed based on recently published scientific literature. Likewise, the industrial implementation and economic feasibility of using NTTs as abiotic elicitors is also discussed. Finally, research needs in this emerging research area are highlighted.

## 2. Wounding Stress as a Tool to Induce the Biosynthesis of Health-Promoting Compounds in Horticultural Crops: Mechanism and Drawbacks in Industrial Practice

**Figure 1:**
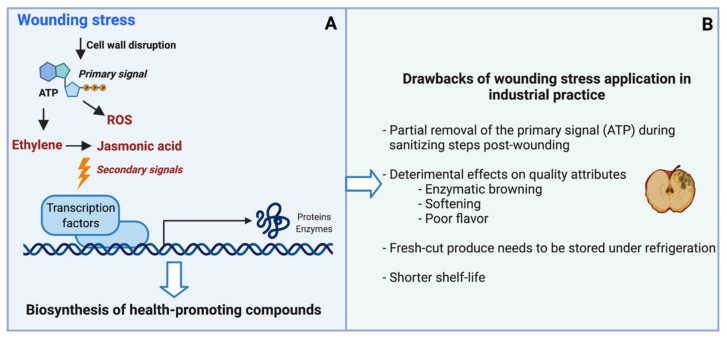
Wounding stress as a tool to induce the biosynthesis of health-promoting compounds in horticultural crops: physiological mechanism involved in stress response, (**A**); drawbacks in industrial practice, (**B**). Abbreviations: ATP, adenosine triphosphate; ROS, reactive oxygen species.

As mentioned in the introduction, applying wounding stress to fruits and vegetables postharvest is an effective strategy to induce the biosynthesis and accumulation of health-promoting compounds. However, wounding stress presents different drawbacks when evaluating the feasibility of its application at industrial scale. To better explain these drawbacks and search for alternative technologies that could emulate the wound response in horticultural crops, the physiological mechanisms governing the wound-induced biosynthesis of secondary metabolites in the plant cell as well as the main drawbacks of wounding stress application as a postharvest technology are described herein and summarized in Figure 1.

As an immediate response to wounding stress, adenosine triphosphate (ATP) is released from the cytoplasm of damaged cells, serving as the primary signal to elicit the wound response [8,16,17]. Subsequently, ATP binds to receptors from intact cells, eliciting the production of secondary stress-signaling molecules such as reactive oxygen species (ROS), ethylene, and jasmonic acid (Figure 1A). These secondary signals trigger the expression of transcription factors, genes, and enzymes related to the biosynthesis of health-promoting compounds [4]. The accumulation of secondary metabolites results from a balance between their biosynthesis and their utilization rates to accomplish a specific physiological function in the plant tissue. For instance, soluble phenolic compounds are biosynthesized in wounded plant cells to serve as precursors for the biosynthesis of lignin or suberin, which prevents water loss in the wounded tissue [18,19,20]. Likewise, glucosinolates in wounded tissue also serve as precursors for isothiocyanate production; these are toxic for insects and protecting plant tissues from pathogen attack [21,22].

The industrial implementation of wounding stress as a technology to produce minimally processed fruits and vegetables with enhanced health benefits presents different drawbacks that need to be addressed (Figure 1B). For instance, as a common practice to ensure the safety of fresh-cut products, the tissue is subjected to washing and sanitizing procedures after cutting. These steps partially remove ATP released from wounded cells, which serves as the primary wound signal (Figure 1A). Therefore, the partial removal of ATP can impede the accumulation of bioactive compounds in the tissue. On this note, sanitizing procedures applied only before peeling and cutting are recommended in order to diminish this drawback [8]. Moreover, wounding stress induces changes in the physiology of horticultural crops, resulting in a loss of quality attributes of fresh produce. For example, cutting fruits and vegetables alters flavor by modifying their acid and sugar content and by inducing synthesis of volatile organic compounds. Furthermore, wounding stress favors the biosynthesis, degradation, and oxidation of secondary metabolites, and induces the depolymerization and degradation of cell membranes, resulting in color changes and texture softening [9]. All of these physiological modifications in the tissue induced by wounding result in a loss of homeostasis and shorter shelf-life of the product. Thus, minimally processed fruits and vegetables need to be commercialized under refrigeration in order to reduce metabolic activity and reduce the loss of quality attributes of the tissue. Furthermore, incorporating preservatives in fresh-cut fruits and vegetables to extend their shelf-life is also a common practice that needs to be reduced through consumer demand for clean labels.

## 3. Scientific Evidence for Non-Thermal Technologies (NTTs) That Emulate Wound-Induced Biosynthesis of Bioactive Compounds in Whole Fruits and Vegetables

As described in the previous section, wounding stress induces high activation of the secondary metabolism of horticultural crops, leading to the accumulation of bioactive compounds. However, the industrial application of wounding to generate fruits and vegetables with enhanced health benefits presents different drawbacks associated with a partial removal of the primary wound signal (ATP) due to sanitizing steps (decreasing elicitation), as well as an increase in oxidative reactions that results in quality loss in the fresh produce. Therefore, it is highly relevant to search for technologies that emulate the wound-induced biosynthesis of bioactive compounds while decreasing the detrimental effects of cutting. In this context, technologies that can increase the health benefits of whole fruits and vegetables are highly valuable and can make for very attractive commercialized functional food products.

The application of NTTs (HHP, US, and PEF) as abiotic elicitors to induce the biosynthesis of bioactive compounds in whole fruits and vegetables has been recently evaluated in different studies. Jacobo-Velázquez et al. [10] proposed that this stress response is elicited through a wound-like response mainly due to the cell membrane disruption induced by NTTs through different driving forces. For PEF and US, cell membrane disruption is produced by an electric potential and by a pressure/temperature gradient in the membrane, respectively. On the other hand, membrane permeability via HHP is induced by a pressure gradient in the plasma membrane [10,23]. The model presented by Jacobo-Velázquez et al. [10] hypothesizes that when membrane permeability is induced, ATP is released from damaged cells and serves as primary signal to induce a wound-like response (Figure 1A). A key aspect to consider is the NTT processing conditions selected, which should be moderate in order to elicit the stress response while preventing plant cell death. In the specific case of HHP, processing conditions should not exceed 100 MPa, while for US, high-intensity treatments with high energy and low frequency (20 to 100 kHz) are adequate for elicitation. Finally, moderate-intensity PEF (MIPEF, 0.5–5 kV cm^−1^, 1–20 kJ kg^−1^) generates reversible damage and elicitation in the plant cell [10].

In the following sections, recent scientific reports describing the effects of HHP, US, and PEF as elicitors to induce the biosynthesis of health-promoting compounds in different whole fruits and vegetables are summarized. Likewise, the effects of NTTs on the quality attributes of whole fresh produce are also described.

### 3.1. High Hydrostatic Pressure (HHP)

The results from previous studies evaluating the effects of HHP treatment on the biosynthesis of health-promoting compounds and the physiological and quality attributes of whole mangoes and carrots are summarized in Table 1.

#### 3.1.1. Mango (*Mangifera indica*)

Hu et al. [24] evaluated the effects of HHP applied at 20, 40, 60, and 80 MPa for 10 min on the biosynthesis and accumulation of carotenoids, phenolics, and ascorbic acid in whole mangoes stored for 16 days at 13 °C. The authors reported that HHP increased the biosynthesis of carotenoids at the transcriptional level, whereas samples treated at 20 MPa presented the highest carotenoid content (43.7% higher than the non-treated samples) during storage. Furthermore, treating mango at 40 MPa resulted in higher ascorbic acid retention, whereas samples treated at 20 MPa presented higher flavonoid content. Similarly, Álvarez-Virrueta et al. [25] and Ortega et al. [26] evaluated the effects of HHP treatment (15, 30, or 60 MPa for 10 or 20 min) on the accumulation of carotenoids and phenolics in whole mangoes stored for 14 days at 25 °C. The authors found that treating whole mangoes at 30 and 60 MPa for 20 min increased the ascorbic acid content by 30.7–46.1% after storage. Likewise, HHP treatment at 15 MPa for 10 min induced a higher accumulation of total carotenoids (100%) and phenolics (41.2%) compared with the control at 14 days of storage. These results indicate that HHP under mild conditions (15 and 20 MPa for 10 min) induced the biosynthesis of carotenoids and phenolics in whole mangoes [24,25,26].

**Table 1:** Effects of high hydrostatic pressure (HHP) on the biosynthesis of health-promoting compounds and the quality and physiological attributes of whole fruits and vegetables.

Horticultural Crop	HHP Processing and Storage Conditions Evaluated	Main Findings		References
		Effects on the Biosynthesis of Health-Promoting Compounds	Effects on Quality and Physiological Attributes	
Mango (*Mangifera* *indica*)	Whole mangoes (cv. Tainong) were subjected to 20, 40, 60, and 80 MPa for 10 min. Samples were stored for 16 days at 13 °C and ~85% relative humidity (RH).	HHP increased carotenoid biosynthesis at the transcriptional level. Samples treated at 20 MPa showed 43.7% higher total carotene content after storage. HHP increased ascorbic acid retention during storage. Samples treated with 40 MPa showed higher ascorbic acid retention. Except for the 40 MPa treatment, HHP-treated samples showed higher flavonoid content. In general, 20 MPa treatment resulted in the highest accumulation of total phenolics and carotenoids, while 40 MPa treated mangoes showed higher levels of ascorbic acid.	HHP treatment reduced respiration rate by 26.62, 20.25, 32.72, and 41.81%, for mangoes treated at 20, 40, 60, and 80 MPa, respectively, compared with the control. HHP treated samples showed higher a * (redness) values during ripening compared with the control. HHP treatment decreased firmness at the initial storage time (1 day). However, after 7 days, no significant difference was observed in firmness values between HHP-treated samples and the control. HHP-treated mangoes showed higher titratable acidity (from days 7 to 16), higher reducing sugar content and higher moisture loss.	[24]
	Whole mangoes (cv. Ataulfo) were treated with HHP at 15, 30, or 60 MPa for 10 or 20 min. Non-treated fruit was used as the control. Samples were stored for 14 days at 25 °C and 85–90% of RH.	HHP treatments at 30 and 60 MPa for 20 min increased ascorbic acid content by 30.7–46.1% compared to the control before storage. HHP treatment at 15 MPa for 10 min induced a higher accumulation of total carotenoids (100%) and phenolics (41.2%) compared with the control at 14 days of storage. Results indicated that low-pressure treatments induced the biosynthesis of nutraceuticals in mango.	HHP did not inhibit the ripening process of mango. At the climacteric peak (9 days), the HHP-treated samples showed a lower respiration rate. Ethylene production was lower in samples treated at 30 and 60 MPa, regardless of the pressurization time (10 or 20 min). HHP-treated mangoes showed lower firmness values during storage, whereas at 14 days, no significant difference was detected between HHP samples and the control. HHP treatment increased moisture loss. At 9 days of storage, the pulp of HHP-treated mangoes showed a more intense orange color than non-treated samples.	[25,26]
Carrot (*Daucus carota*)	Whole carrots were treated at 60 or 100 MPa for the come-up time (CUT). Samples were stored for 3 days at 15 °C.	Immediately after HHP application, carrots treated at 100 MPa showed an increase of free (5-*O*-caffeoylquinic acid, 63.9% and 3,4-di-*O*-feruloylquinic acid, 228.6%) and bound (p-coumaric acid, 82.6%) phenolics. On day 1, the 60 MPa samples showed accumulation of 4,5-di-*O*-caffeoylquinic acid (60.2%) and isocoumarin (98.9%), whereas the 100 MPa samples presented increases of chlorogenic acid (291.2%) and 3,4-di-*O*-feruloylquinic acid (466.1%). On day 2, an increase in bound phenolics (rutin, 85.5% and p-coumaric acid, 214.7%) was observed in samples treated at 60 MPa. On day 3, the 100 MPa samples presented higher quercetin (371.2%) content.	On day 2, the 60 MPa and 100 MPa samples showed 380.2% and 139.7% higher phenylalanine-ammonia lyase (PAL) activity, respectively, than the control. On day 3, the 60 MPa samples presented 212% higher PAL activity than the control, whereas no significant difference was observed between the 100 MPa samples and the control. During storage, higher ethylene production and respiration rate were detected in the 60 and 100 MPa samples compared with the control.	[27]
	HHP treatments were applied for the CUT as a single pulse or multi-pulse (2P, 3P, and 4P). In addition, a single sustained treatment (5 min) was applied at 60 or 100 MPa. Samples were stored for 48 h at 15 °C.	Immediately after HHP treatment, the extractability of phenolics increased by 66.65% and 80.77% in 3P 100 MPa and 4P 60 MPa samples, respectively. After storage, CUT 60 MPa samples accumulated free (163.05%) and bound (36.95%) phenolics. Total xanthophylls increased by 27.16% after CUT 60 MPa treatment, whereas no changes were observed after storage.	The authors did not report quality characteristics and physiological measurements of the samples.	[28]

The effect of HHP treatments on the quality and physiological attributes of whole mangoes was also reported in the studies described above [24,25,26]. Pressurization did not inhibit the ripening process of mango, but reduced the respiration rate by 26.62, 20.25, 32.72, and 41.81% for the whole tissue treated at 20, 40, 60, and 80 MPa, respectively, as compared with the control. In addition, at the climacteric peak, whole mangos treated with HHP showed a lower respiration rate [24]. Likewise, HHP-treated mangoes showed higher a* (redness) values during ripening than the control. Moreover, pressure treatments decreased firmness at the initial storage time (1 day), whereas after 7 days of storage no significant difference was observed between HHP treated samples and the control. The immediate decrease in firmness was attributed to the loss of turgor induced by vacuolar content leakage upon pressurization [24]. Likewise, the increase in firmness of HHP-treated samples during storage was attributed to an increase in pectinmethylesterase (PME) activity, which upon contact with the substrate results in the formation of pectates and pectin gelation, promoting firmness [24]. Pressurized whole mangoes showed higher titratable acidity (from days 7 to 16), higher reducing sugar content, and higher moisture loss during storage [24]. Furthermore, ethylene production was lower in whole mangoes treated at 30 and 60 MPa for 10 or 20 min. Finally, during storage, the pulp of HHP treated mangoes showed a more intense orange color, associated with the biosynthesis of carotenoids during ripening [25,26,29].

#### 3.1.2. Carrot (*Daucus carota*)

Regarding carrots, Viacava et al. [27] evaluated the effects of HHP treatment and storage time (15 °C for 3 days) on the biosynthesis of health-promoting compounds and physiological attributes of the whole tissue. Carrots were treated at 60 or 100 MPa for the time needed to reach the desired pressure (come-up time, CUT). Immediately after HHP processing, an increase of free (5-*O*-caffeoylquinic acid (63.9%) and 3,4-di-*O*-feruloylquinic acid (228.6%)) and bound (*p*-coumaric acid (82.6%)) phenolics was detected. Furthermore, whole carrots treated at 60 MPa and stored for 1 day showed accumulation of the free phenolic 4,5-di-*O*-caffeoylquinic acid (60.2%), whereas samples treated at 100 MPa showed increases of chlorogenic acid (291.2%) and 3,4-di-*O*-feruloylquinic acid (466.1%). On day 2, samples treated at 60 MPa showed an increase in bound phenolics (rutin (85.5%) and *p*-coumaric acid (214.7%)). Finally, at the end of the storage period (day 3), samples treated at 100 MPa showed higher quercetin content (371.2%) than the control. Similarly, Viacava et al. [28] evaluated the effect of HHP treatments (60 or 100 MPa) applied for a single sustained pulse (5 min) or the CUT as a single pulse (P) or multi-pulse (2P, 3P, and 4P) on the accumulation of phenolic compounds and carotenoids after 48 h of storage at 15 °C. The authors reported that immediately after HHP treatment, the extractability of phenolics increased by 66.65% and 80.77% in 3P 100 MPa and 4P 60 MPa samples, respectively. Likewise, whole carrots treated for the CUT 60 MPa accumulated free (163.05%) and bound (36.95%) phenolics after storage. Furthermore, the content of total xanthophylls increased by 27.16% immediately after CUT 60 MPa treatment, whereas no changes in carotene concentration were observed after storage.

Regarding the effects of HHP treatment on the physiological attributes of carrots, after 2 days of storage (15 °C), whole carrots pressurized at 60 MPa and 100 MPa showed 380.2% and 139.7% higher phenylalanine-ammonia lyase (PAL) activity, respectively, as compared with the control, whereas at day 3, samples treated at 60 MPa showed 212% higher PAL activity than the control, with no significant difference observed in PAL activity between the 100 MPa samples and the control. HHP-induced PAL activation confirmed that pressure induced the biosynthesis of phenolic compounds in whole carrots. During storage, samples treated at 60 and 100 MPa showed higher ethylene production and respiration rates than the control [27]. These results are in contrast with the results reported for mangoes, where HHP reduced respiration and ethylene production, suggesting that the HHP effect on the physiological attributes of fresh produce is tissue-dependent.

One of the main physiological differences between carrots and mangoes that could result in a different postharvest behavior as a response to HHP is that carrot is a non-climacteric vegetable, whereas mango is a climacteric fruit. Climacteric and non-climacteric classification of fruits and vegetables depends on their ripening patterns and the presence of a burst in ethylene production and respiration rate during postharvest, also known as the climacteric peak [9]. Results from the literature cited herein indicate that HHP affects climacteric and non-climacteric produce differently. Interestingly, if a wound-like response is expected due to HHP application in mango, an accelerated climacteric peak and an associated increased loss of quality characteristics should be occurring in the pressurized tissue. However, in contrast with wounding, HHP reduced ethylene production and respiration of the fruit, preserving its quality characteristics during storage and overcoming this major drawback of wounding stress application.

### 3.2. Ultrasound (US)

The effects of US treatment on the biosynthesis of health-promoting compounds and physiological attributes of whole fruits and vegetables such as lettuce, strawberry, carrot, and broccoli have been previously reported (Table 2). Furthermore, the application of US has also been explored as a pretreatment in seeds to improve sprouting indexes and improve the content of health-promoting compounds and the quality characteristics of sprouts commercialized as a ready-to-eat vegetable (Table 2).

#### 3.2.1. Lettuce (*Lactuca sativa*)

Yu et al. [30] evaluated the effects of US treatment (25 kHz) at an acoustic power density of 26 W/L for 1–3 min on whole leaf lettuce. The US-treated samples were stored at room temperature for 150 h. Immediately after US treatment, no significant difference in total phenolics was detected between the control and the processed lettuce. At 60 h of storage, samples treated for 1 min of US showed 22.50% higher phenolics than the control, which was accompanied by an increase in PAL activity, confirming that US induced the activation of the phenylpropanoid metabolism. Samples treated with US showed higher firmness during storage, while the color was not affected [30].

#### 3.2.2. Strawberry (*Fragaria x ananassa*)

Strawberries were treated with US (33 kHz, 60 W, 25 °C) for different times (0, 10, 20, 30, 40, and 60 min) and stored for 15 days at 4 °C [31]. US-treated strawberries showed higher ascorbic acid retention in the fruit, with decreases of 54.68, 36.68, 35.57, and 32.20% observed for treatment times of 0, 10, 20, and 30 min, respectively. US increased total phenolics on day 1. As the US time increased from 0 to 40 min, a significant increase (7.91%) in total phenolics was quantified. However, 60 min US treatment decreased phenolic content by 7.91%. Except for 60 min US treatment, total phenolics increased in both US treated samples and the control.

Regarding the effect of HHP on the physiological and quality attributes of whole strawberries, at day 15 an increase of 13.84, 4.32, 3.20, 3.50, 2.93, and 13.88% in pH value was detected in samples subjected to US for 0, 10, 20, 30, 40, and 60 min, respectively. At day 15, total soluble solids increased by 24.66, 21.43, 18.23, 17.07, 18.84, and 30.05% for US treatment of 0, 10, 20, 30, 40, and 60 min, respectively. Immediately after treatment, color change (ΔE*) values increased by 4.61, 5.25, 6.16, 7.94, and 11.29% for US treatments of 10, 20, 30, 40, and 60 min, respectively. Furthermore, US-treated strawberries showed better color retention during storage, and fruit firmness was better retained in US (20–30 min) treated samples. Microbial load also decreased with US treatment. Interestingly, as observed for mango treated with HHP, strawberries subjected to US showed improved texture during storage. This observation suggests that US could also activate PME, likely by promoting contact between substrate and enzyme, leading to the production of pectates and pectin gelation.

**Table 2:** Effects of ultrasound (US) on the biosynthesis of health-promoting compounds and on the quality and physiological attributes of whole fruits and vegetables.

Horticultural Crop	US Processing and Storage Conditions Evaluated	Main Findings		References
		Effects on the Biosynthesis of Health-Promoting Compounds	Effects on Quality and Physiological Attributes	
Romaine lettuce (*Lactuca* *sativa*, var. *longifolia*)	Whole leaf lettuce was treated with US (25 kHz) at an acoustic power density of 26 W/L for 1–3 min. Samples were stored at room temperature for 150 h.	Immediately after treatment, no significant difference in total phenolics was detected between the control and US-treated lettuce. At 60 h of storage, samples treated for 1 min of US showed 22.50% higher phenolics than the control. Lettuce treated with US for 2 and 3 min did not show significant increases in phenolics compared with the control. After 90 h, no significant difference in phenolic content was detected between the control and US-treated samples.	In the first 30 h, no phenylalanine ammonia-lyase (PAL) activation was detected due to US treatment. At 60 h, samples treated with 2 and 3 min of US showed the highest PAL activity. US-treated lettuce showed higher firmness (maximum force, N) than the control (water washed) immediately after treatment and during storage. No significant difference was detected in the color characteristics of lettuce between treatments.	[30]
Strawberry (*Fragaria x ananassa*, cv. chandler)	US treatment (33 kHz, 60 W, 25 °C) was applied to strawberries for different times (0, 10, 20, 30, 40, and 60 min). Samples were stored for 15 days at 4 °C.	US treatments (10–30 min) increased ascorbic acid retention in the tissue, where decreases of 54.68, 36.68, 35.57, and 32.20% were observed for treatment times of 0, 10, 20, and 30 min, respectively. US treatments of 40 and 60 min resulted in a decrease in the retention of ascorbic acid. US increased total phenolics on day 1. As the US time increased from 0 to 40 min, a significant increase (7.91%) in total phenolics was quantified. However, 60 min US treatment decreased phenolic content by 7.91%. Except for 60 min US treatment, total phenolics increased in US-treated samples as well as in the control.	On day 15, an increase of 13.84, 4.32, 3.20, 3.50, 2.93, and 13.88% in pH value was detected in samples subjected to US for 0, 10, 20, 30, 40, and 60 min. respectively. At day 15, total soluble solids increased by 24.66, 21.43, 18.23, 17.07, 18.84, and 30.05% for US treatment of 0, 10, 20, 30, 40, and 60 min, respectively. Immediately after treatment, the value of ΔE* increased by 4.61, 5.25, 6.16, 7.94, and 11.29% for 10, 20, 30, 40, and 60 min US treatments, respectively. US-treated strawberries had better color retention during storage. Fruit firmness was better retained in US (20–30 min)-treated samples. Microbial load also decreased with US treatment.	[31]
Carrot (*Daucus carota*)	Whole carrots were treated with US (frequency 24 kHz, amplitude 100 mm) for 5 min at 20 °C. Samples were stored at 20 °C for 3 days.	As an immediate response to US, carrots showed 21.1% higher levels of total carotenoids as compared with the control. After storage, carotenoid content decreased. However, US-treated samples showed a lower decrease in carotenoid (−7.6%) than the control (−16.4%). US treatment induced an immediate decrease in total phenolic content (−62.5%) compared with the control. After 3 days of storage, phenolic content in US-treated samples increased by 129.2%, whereas for control samples the phenolic content did not change. Chlorogenic acid was the main phenolic compound that increased in US-treated whole carrots, which showed 41.8% higher content at 1 day of storage than the control.	On day 3 of storage, US-treated whole carrots showed the highest isocoumarin content, 164.0% higher than the control and 240.8% higher than the control before storage. Isocoumarin exerts a bitter flavor in carrots at concentrations of 200 mg/kg [LaFuente et al. 1996]. However, the levels detected in sonicated carrots were below the threshold of sensory perception (40 mg/kg). US-treated samples showed a 2.04-fold increase in expression of the *PAL* gene immediately after treatment. During the first 0.5 days of storage, PAL activity increased in US-treated carrots, showing 64.8% higher activity than the control before storage. US induced an immediate increase in respiration rate. US-treated whole carrots showed 27.9%, 66.0%, and 162.0%, higher levels of volatile organic compounds (indicators of ethylene production) at 0.5, 1.5, and 2 days of storage, respectively.	[32]
Broccoli (*Brassica oleracea* L. var. *Italica*, cv. Tlaloc^®^)	Broccoli florets were treated with US (20 min, frequency 24 kHz, amplitude 100 μm). US-treated broccoli florets were subjected to exogenous methyl jasmonate (MJ, 250 ppm) and/or ethylene (ET, 1000 ppm) during storage. Samples were stored for 3 days at 15 °C.	As an immediate response to US treatment, the extractability of glucosinolates (glucoraphanin (795%), glucobrassicin (78.6%), and 4-hydroxy glucobrassicin (153%)) and phenolics (1-sinapoyl-2-feruloylgentiobiose (57.23%)) was increased. At 3 days, samples treated with US and MJ showed the highest accumulation of gluconasturtiin (755.9%), neoglucobrassicin (232.8%), 4-hydroxy glucobrassicin (187.1%), glucoerucin (111.92%), 1,2,2-trisinapoylgentiobiose (136.7%), 3-*O*-caffeoylquinic acid (73.4%), and 1-sinapoyl-2-ferulolylgentiobiose (56.0%) as compared with the control. Ascorbic acid content decreased during storage. However, US and exogenous phytohormones (MJ and ET) in combination reduced ascorbic acid degradation.	The authors did not report quality characteristics and physiological measurements of the samples.	[33]
Common bean (*Phaseolus* *vulgaris*, cv. Kabulengeti) sprouts	Seeds were treated with US at different power (0, 180, and 360 W) and time (0, 30, 45, and 60 min) before sprouting. Seeds were sprouted in darkness (at 25 °C) for 96 h.	At 96 h of sprouting, the accumulation of total phenolic acids, flavonoids, and anthocyanins increased with the intensity of the US treatment. Total phenolic acids, flavonoids, and anthocyanins were 1065.27%, 559.8%, and 1052.9%, higher at 96 h of sprouting, respectively, in seeds treated with 360 W (60 min) as compared with the control.	US treatments decreased radicle emergence time, increased radicle length (72.37%), and induced the maximum hypocotyl growth (60.44%) when comparing 360 W (60 min) with the control at 96 h of sprouting time. US treatment enhanced the sprouting percentage, sprouting index and vigor of the samples, improving the quality characteristics of common bean sprouts. Hydrogen peroxide production and the activity of catalase, glutathione peroxidase, PAL, and tyrosine ammonia-lyase were also higher during sprouting of seeds treated with 360 W for 60 min compared with the control.	[34]
Soybean (*Glycine max* L. cv. Dongnong 48) sprouts	Soybean seeds were treated with US at different power levels (0, 100, 200, and 300 W, 40 kHz, for 30 min). The seeds were germinated at 30 °C for 5 days in darkness.	Gamma-aminobutyric acid (GABA) accumulated in higher concentrations in soybean sprouts as the US power level increased, with sprouts from seeds treated at 300 W for 30 min showing 43.5% higher GABA content compared with the control. US pretreatment in seeds (300 W, 30 min) generated soybean sprouts with lower content of daidzin (−79.62%) and genistin (−70.95%), and higher content of daidzein (39.13%) and genistein (94.91%) as compared with the control sprouts.	The germination rate and average length of sprouts increased by 18.07% and 20.41%, respectively, after US (300 W, 30 min) as compared with the control. US pretreatment (300 W, 30 min) reduced lipoxygenase (LOX) activity by 36.22 to 55.57% (depending on the LOX isomer), resulting on sprouts with improved odor and flavor. Sprouts obtained from seeds treated at 300 W for 30 min showed the highest decrease in IgE-binding potency (51.39%), and resulted in soybean sprouts with 98.78% less trypsin inhibitor.	[35]
Peanut (*Arachis* *hypogaea* L.) sprouts. Three cultivars were evaluated (Fuhua12, Fuhua 18, and Baisha 1016)	Peanut seeds were treated with US at 28, 45, and 100 kHz for 15, 20, and 30 min. Seeds were germinated under dark at 28 °C and 90% relative humidity for 5 days.	Resveratrol content of sprouts increased with prolonged US and soaking times, showing optimum conditions at 20 min and 6 h, respectively. Fuhua cultivar treated with US (100 kHz, 20 min) presented an increase in resveratrol content (980.1%) after sprouting compared with the control.	Germination rate was increased for seeds treated with 100 kHz ultrasonic waves. The allergic protein content completely decreased at 3 days of germination when treating the seeds with 100 kHz for 20 min before germination.	[36]

#### 3.2.3. Carrot (*Daucus carota*)

Cuellar-Villarreal et al. [32] evaluated the effect of US (frequency 24 kHz, amplitude 100 mm) applied for 5 min at 20 °C, on the phenolic and carotenoid content in carrots immediately after treatment and after storage (20 °C for 3 days). As an immediate response to US, carrots showed 21.1% higher levels of total carotenoids as compared with the control. After storage, carotenoid content decreased in carrots. However, US-treated samples showed a lower decrease in carotenoid (−7.6%) than the control (−16.4%). US treatment induced an immediate decrease in total phenolic content (−62.5%) compared with the control. Moreover, after 3 days of storage, phenolic content in US-treated carrots increased by 129.2%, whereas for control samples the phenolic content did not change. Chlorogenic acid was the main phenolic compound that increased in US-treated whole carrots, which showed 41.8% higher content at 1 day of storage than the control. Interestingly, US-treated whole carrots at 3 days of storage showed the highest isocoumarin accumulation, 164.0% higher than the control and 240.8% higher than the control before storage. It is well known that isocoumarin exerts bitter flavor in carrots at 200 mg/kg [37]. However, the levels detected in sonicated carrots were below the threshold for sensory perception (40 mg/kg). The US-induced biosynthesis of phenolic compounds in carrots was confirmed by the immediate increase (2.04-fold increase) in *PAL* gene expression detected after treatment, which was accompanied by an increase in PAL enzymatic activity during the 0.5 days of storage, which was 64.8% higher as compared with the control before storage. US induced an immediate increase in respiration rate and higher levels of volatile organic compounds compared with the control.

#### 3.2.4. Broccoli (*Brassica oleracea*)

The US-induced accumulation of health-promoting compounds has also been evaluated in broccoli [33]. The authors treated broccoli florets with US (20 min, frequency 24 kHz, amplitude 100 μm) and stored (3 days at 15 °C) the ultrasonicated samples in the presence of exogenous methyl jasmonate (MJ, 250 ppm) and/or ethylene (ET, 1000 ppm). As an immediate response to US treatment, the extractability of glucosinolates (glucoraphanin (795%), glucobrassicin (78.6%), and 4-hydroxy glucobrassicin (153%)) and phenolics (1-sinapoyl-2-feruloylgentiobiose (57.23%)) was increased. Furthermore, at 3 days of storage, broccoli florets treated with US and MJ showed the highest accumulation of gluconasturtiin (755.9%), neoglucobrassicin (232.8%), 4-hydroxy glucobrassicin (187.1%), glucoerucin (111.92%), 1,2,2-trisinapoylgentiobiose (136.7%), 3-*O*-caffeoylquinic acid (73.4%), and 1-sinapoyl-2-ferulolylgentiobiose (56.0%) as compared with the control. Likewise, ascorbic acid content decreased during storage of broccoli florets; however, the combined application of US, MJ, and ET reduced ascorbic acid degradation. These results indicate that the combined application of US treatment and exogenous phytohormones resulted in an effective strategy to induce the biosynthesis of glucosinolates and retain vitamin C content during storage.

#### 3.2.5. Common Bean (*Phaseolus vulgaris*) Sprouts

The application of US has also been explored as a pretreatment in seeds to improve the content of health-promoting compounds and quality characteristics of sprouts. In this context, common bean seeds were treated with US at different power (0, 180, and 360 W) and time (0, 30, 45, and 60 min) before sprouting [34]. US-treated bean seeds and the control were sprouted in darkness (at 25 °C) for 96 h in an incubator. At 96 h of sprouting, the accumulation of total phenolic acids, flavonoids, and anthocyanins increased with the intensity of the US treatment. Total phenolic acids, flavonoids, and anthocyanins were 1065.27%, 559.8%, and 1052.9% higher at 96 h of sprouting, respectively, in seeds treated with 360 W (60 min) as compared with the control. US treatments decreased radicle emergence time, increased radicle length (72.37%), and induced the maximum hypocotyl growth (60.44%) when comparing 360 W (60 min) with the control at 96 h of sprouting time. Sprouting percentage was enhanced by US treatment, with seeds treated with 360 W for 60 min showing the highest values at 36 h of sprouting. US enhanced the sprouting index and vigor of samples, improving the quality characteristics of common bean sprouts. Hydrogen peroxide production and the enzymatic activity of catalase, glutathione peroxidase, PAL, and tyrosine ammonia-lyase were also higher during sprouting of seeds treated with 360 W for 60 min as compared with the control, suggesting that US induced the biosynthesis of phenolic compounds through a ROS-mediated mechanism.

#### 3.2.6. Soybean (*Glycine max*) Sprouts

Soybean seeds were treated with US at different power levels (0, 100, 200, and 300 W, 40 kHz, for 30 min). The seeds were germinated at 30 °C for 5 days in darkness [35]. Gamma-aminobutyric acid (GABA) accumulated in higher concentrations in soybean sprouts as US power levels increased, with sprouts from seeds treated at 300 W for 30 min showing 43.5% higher GABA content compared with the control. US pretreatment in seeds (300 W, 30 min) generated soybean sprouts with a lower content of daidzin (−79.62%) and genistin (−70.95%), and higher content of daidzein (39.13%) and genistein (94.91%) compared with the control sprouts, indicating that US application before germination of soybean seeds induces the activation of glucosidases during sprouting, generating isoflavone aglycones.

The germination rate and average length of soybean sprouts increased by 18.07% and 20.41%, respectively, after US (300 W, 30 min) compared with the control. US pretreatment (300 W, 30 min) generated a reduction in lipoxygenase (LOX) activity by 36.22% and 55.57% (depending on the LOX isomer), resulting in sprouts with improved odor and flavor. Sprouts obtained from seeds treated at 300 W for 30 min showed the highest decrease in IgE-binding potency (51.39%), indicating that US treatment induced the degradation of allergens into peptides and amino acids. US pretreatment at 300 W resulted in soybean sprouts with 98.78% less trypsin inhibitor.

#### 3.2.7. Peanut (*Glycine max*) Sprouts

Yu et al. [36] evaluated the effect of US (28, 45, and 100 kHz for 15, 20, and 30 min) as a pretreatment for germination in peanut seeds and determined the resveratrol content during germination for 5 days (Table 2). The authors reported that the resveratrol content of sprouts increased (980.1%) with prolonged US and soaking times, showing optimum conditions at 20 min and 6 h, respectively. Germination rate increased for seeds treated with 100 kHz ultrasonic waves. The allergic protein content completely decreased at 3 days of germination time by treating the seeds with 100 kHz for 20 min prior to germination. Thus, in addition to increasing the content of health-promoting compounds, US pretreatment decreases the natural antinutrient content in the seeds.

### 3.3. Pulsed Electric Fields (PEF)

The effect of moderate-intensity pulsed electric fields (MIPEF) on the accumulation of health-promoting compounds has been evaluated in fruits and vegetables such as apple [38], tomato [39,40,41], and carrot [42,43] (Table 3).

#### 3.3.1. Apple (*Malus domestica*)

Soliva-Fontuny et al. [38] treated whole apple fruits at 0.4–2 kV cm^−1^ using 5–35 monopolar pulses of 4 μs at a frequency of 0.1 Hz, corresponding to a specific energy input of 0.008–1.3 kJ kg^−1^. The apples were stored at 4 and 22 °C for 48 h. MIPEF treatment (0.008 kJ kg^−1^) induced an increase in total phenolic (13%) and flavan-3-ol (92%) contents in fruits stored for 24 h at 22 °C, and in flavonoids (58%) in samples stored at 4 °C. The authors did not report quality characteristics or physiological measurements of MIPEF-treated apples.

#### 3.3.2. Tomato (*Lycopersicon esculentum*)

Tomato fruits were subjected to different electric field strengths (from 0.4 to 2.0 kV cm^−1^) and number of pulses (from 5 to 30), and the content of phenolics and carotenoids was detected after storage (4 °C for 24 h). Except for the 2 kV/cm treatment, MIPEF-treated tomatoes showed a higher phenolic content after 24 h of treatment compared to the control [39,40]. This increase ranged from 6.6% (5 pulses at 0.4 kV/cm) to 44.6% (30 pulses at 1.2 kV/cm). Accumulation of lycopene was detected after storage of MIPEF-treated tomato fruit, which ranged from 0.6% (18 pulses at 2 kV/cm) to 31.8% (5 pulses at 1.2 kV cm^−1^) as compared with non-treated tomato. Likewise, MIPEF treatments at 1.2 kV cm^−1^ and 30 pulses resulted in the highest accumulation of caffeic acid-*O*-glucoside (170%), caffeic acid (140%), and chlorogenic acid (152%). MIPEF treatments at 1.2 kV cm^−1^ and five pulses resulted on the highest accumulation of α-carotene (93%), 9-*cis*-lycopene (94%) and 13-*cis*-lycopene (140%), respectively. The authors did not report quality characteristics and physiological measurements of MIPEF-treated tomato fruits [40].

In another study, González-Casado et al. [41] subjected tomato fruits at different electric field strengths (40, 120, and 200 kV m^−1^) and number of pulses (5, 18, and 30 pulses). Specific energy input ranged from 0.02 to 2.31 kJ kg^−1^. PEF-treated tomato fruits were stored at 4 °C for 24 h. The highest increase in carotenoid content (50%) was achieved in tomato fruits treated with an energy input of 2.31 kJ kg^−1^ (200 kV m^−1^ and 30 pulses). MIPEF-treated tomatoes showed a significant increase in the respiration rate of tomato fruit. Ethylene concentration was higher (53%) in fruits subjected to the lowest electric field strength, whereas a further increase in PEF intensity resulted in depletion of ethylene concentration. This result suggests that PEF could induce ethylene biosynthesis in even the mildest conditions. Acetaldehyde synthesis was induced when tomatoes were subjected to energy inputs higher than 0.38 kJ kg^−1^, indicating that the tissue was undergoing plant cell death. Higher treatment intensities resulted in a more pronounced softening effect, with a reduction in the firmness values of 80% observed in the fruits treated with the most intense PEF conditions. Total soluble solids and pH values increased with treatment intensity. Accumulation of sugars in PEF-treated tomatoes was attributed to a stress response in the tissue related to osmoregulation to restore plant cell activity as well as with a PEF-induced increase in the ripening process of the fruit [41]. Furthermore, the authors associated the increase in pH with increased respiration induced by PEF, since organic acids are used as substrates.

#### 3.3.3. Carrot (*Daucus carota*)

Regarding carrots, López-Gámez et al. [42] treated the whole tissue with different electric field strengths (0.8, 2.0, and 3.5 kV cm^−1^) and number of pulses (5, 12, and 30). Samples were stored for 48 h at 4 °C, and the effects of PEF on phenolic content, cell viability, texture softening, and color of carrots were determined (Table 3). The most significant increase in phenolic content was observed at 24 h, after applying five pulses of 3.5 kV cm^−1^ (39.5%) and 30 pulses of 0.8 kV cm^−1^ (40.1%). A correlation between the specific energy input and cell viability was found. After applying 3.5 kV cm^−1^, viability decreased by 87.5–79.4%. At 24 h, whole carrots treated with five pulses of 3.5 kV cm^−1^ and 30 pulses of 0.8 kV cm^−1^ showed texture softening with color preservation.

Similarly, López-Gámez et al. [43] processed whole carrots in a PEF batch system with five pulses of 350 kV m^−1^ (580 ± 80 J kg^−1^) and evaluated the treatment effect on the phenolic profile, respiration rate, and color during storage (36 h at 4 °C) of the tissue. Immediately after PEF treatment, the whole carrots showed a decrease in *p*-coumaric acid (42.3%), protocatechuic acid (−78.1%), and ferulic acid (−56.3%). The maximum accumulation of phenolics in whole carrots was reached after 24 h of PEF treatment, showing a significant increase in total phenolics (80.2%), chlorogenic acid (74.9%), ferulic acid (52.2%), and *p*-OH-benzoic (94.7%) acid as compared with the control. At 36 h of storage, a decrease in phenolic content was observed. The application of PEF induced an immediate increase in respiration rate after treatment. From 12 to 36 h, PEF-treated carrots showed between 123–164% more CO_2_ production than untreated carrots. PEF did not induce an immediate increase in production of volatile organic compounds in whole carrots. However, at 12 h of storage, samples treated with PEF generated higher amounts of acetaldehyde (7 pg kg^−1^ s^−1^), ethanol (68 ng kg^−1^ s^−1^) and ethylene (50 ng kg^−1^ s^−1^), whereas these volatiles were not detected in untreated carrots. The authors attributed the presence of acetaldehyde and ethanol to PEF-induced anaerobic metabolism related to structural damage and intracellular content leakage. On the other hand, the increase of ethylene was associated with serving as a stress signaling molecule that induces the activation of secondary metabolism [43].

PEF application delayed the peak of maximum peroxidase enzymatic activity for 12 h. Pectin methylesterase (PME) activity increased during the first 12 h of storage in the control, whereas PEF induced an immediate increase (164%) in enzyme activity, which remained stable for the following storage time. Polygalacturonase (PG) activity immediately decreased by 31–32% after treatment. PAL activity in untreated carrots remained stable during storage, whereas PEF-treated samples showed a constant increase in PAL activity, showing the highest increase (153%) at the end of the storage time. As earlier described, PME strengthens vegetable tissues through cross-linking between pectin chains, whereas PG activity is related to solubilization of pectic substances and softening in many fruits and vegetables. Thus, the results indicate that a PEF-induced increase of PME activity and an induced decrease of PG generates increased cell-wall rigidity in carrots [43].

## 4. Industrial Implementation and Economic Feasibility of Using Non-Thermal Technologies as Tools to Enhance the Content of Health-Promoting Compounds in Whole Fruits and Vegetables

For the last 20 years, NTTs have come a long way in technological development, process optimization, industrial applicability, and consumer acceptability. HHP and PEF have become widely used processing stages on industrial scales [44]. On the other hand, US application in food and beverage processing is relatively new compared to HHP and PEF. Although there are some uses of US on a large scale, there is still work to do to achieve the same acceptability and industrial applicability as other NTTs. Nevertheless, these three NTTs have proven to achieve enzyme and microbial inactivation, enhance shelf-life, and preserve nutritional and nutraceutical properties in foods and beverages. Furthermore, they may be used to enhance extractability and bioavailability in food matrixes [14].

Table 4 presents a qualitative comparison between HHP, PEF, and US processing technology. Each technology is assessed in terms of scaling-up feasibility and cost of operation/maintenance. As mentioned before, HHP is a much more mature technology compared to PEF and US, as it has been studied and optimized for more than 35 years [45,46]. This context is relevant when comparing these technologies. It can be expected that some of the limitations or drawbacks associated with PEF and US will lessen as the technology develops further. All three technologies can process in batch or continuous/semi-continuous mode. Furthermore, as described in the previous section, all of them can process whole fruits and vegetables.

As mentioned above (Table 1, Table 2 and Table 3), NTTs can also be used as a tool to elicit the biosynthesis of nutraceutical compounds in whole fruits and vegetables, enhancing their health-promoting properties. This elicitation is promoted using process conditions that activate the secondary plant metabolism without significantly affecting quality attributes (texture, flavor, color, etc.). HHP, PEF, and US processing conditions to induce stress responses on whole fruits and vegetables are milder than those needed for achieving microbial or enzyme inactivation in processed foods. These milder processing conditions may represent an additional advantage in terms of energy expenditure and equipment maintenance. The processing conditions shown in Table 1, Table 2 and Table 3 can be applied at pilot-plant and industrial scales to obtain whole fresh fruits (i.e., apples, mangoes, tomatoes, and strawberries) and vegetables (i.e., carrots, broccoli, and lettuce) with enhanced levels of health-promoting compounds such as carotenoids, phenolics, and glucosinolates, among others.

Consumer awareness of the benefits of consuming fruits and vegetables meeting desirable quality attributes, safety requirements, and high nutraceutical value increases daily. In such a way, having whole fresh produce with enhanced health-promoting properties is undoubtedly of interest to most consumers. Regarding the economic point of view, it is essential to reflect on how NTT processing may affect product price once in the market. How much would it cost to process whole carrots with HHP to increase their content of health-promoting compounds? Or mangoes? In order to calculate the processing cost, it is necessary to consider aspects such as the energy demand of the equipment (kWh), the cost of all inputs needed (such as the water for the treatment chamber), and the cost of maintenance (estimated to be about 5% of the total inversion cost of the equipment annually, as a rule of thumb). It is also necessary to define a scenario.

In this regard, let us suppose that the goal is to use HHP to process 30 tons of whole carrots per day in order to enhance their health-promoting properties. HHP equipment throughput at industrial scales typically ranges from 200 to 3500 kg/h. Given the processing goal, equipment with an average throughput of 1500 kg/h would be adequate (20 h processing time). Such HHP equipment would have an approximate annual maintenance cost of USD 500,000 and an approximate power requirement of 230 kWh. HHP processing of 30 tons of whole carrots per day would require about 30 m^3^ (1059 ft^3^) of water (the total volume of the chamber minus the volume occupied by the product); this technical information has been taken or estimated using commercial literature from an HHP equipment supplier [47].

In this way, the cost for HHP processing of 30 tons of carrots per day (20 h processing time) can be estimated, considering approximate electricity and water costs for industrial consumers in the USA, as:

- Energy cost: 230 kWh ∗ USD 0.067/kWh ∗ 20 h/day = USD 308.20/day
- Water cost: 1059 ft^3^/day ∗ USD 0.011/ft^3^ = USD 11.65/day
- Maintenance cost: USD 500,000/year ∗ 0.0027 years/day = USD 1350/day
- Estimated HHP processing cost for 30 tons of carrots = USD 1669.85/day

For one kg of HHP-processed whole carrot, the cost is USD 0.056. In the USA, the average price of one kg of fresh carrot is USD 1.19. Adding the HHP processing cost to the average commercial price of carrots gives USD 1.246 per kg, or a 4.71% increase. HHP-processed whole carrots with enhanced health-promoting properties could be easily commercialized at a price 15–30% higher than non-processed carrots, validating the economic feasibility of the process. This same analysis could be done with other NTT such as PEF and US. A wide variety of fruits and vegetables (those included in Table 1, Table 2 and Table 3, as well as many others) may be feasible for large-scale processing using NTT to enhance their health-promoting properties, while preserving their quality attributes. These are exciting times in the optimization and development of new NTT. This technology, used under abiotic stress conditions, may soon constitute a widely-used strategy for enhancing whole fruits and vegetables at a commercial (pilot-plant and industrial) scale.

## 5. Further Research Needs

**Figure 2:**
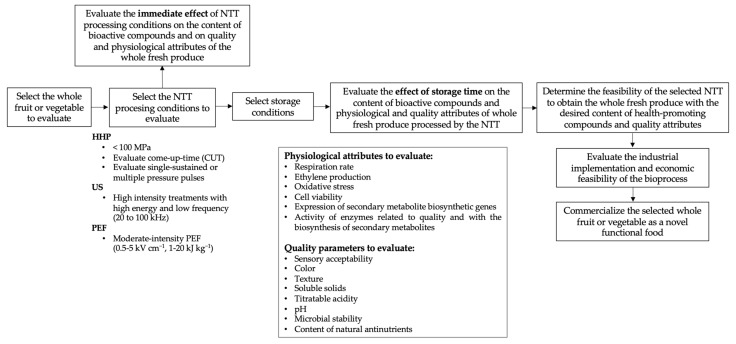
Key aspects to consider when determining the feasibility of an NTT to enhance the content of health-promoting compounds in whole fruits and vegetables while preserving quality attributes. More fundamental knowledge is needed to advance the industrial application of NTTs as abiotic elicitors. Abbreviations: HHP, high hydrostatic pressure; US, ultrasound; PEF, pulsed electric fields; NTT, non-thermal technology.

As described in the previous sections, some scientific research is being performed to evaluate the feasibly of using NTTs as abiotic elicitors in order to increase the content of bioactive compounds in whole fruits and vegetables. However, most of the recent scientific reports are focused on studying the effects of NTTs on either the accumulation of specific secondary metabolites or on the quality and physiological parameters of the crop under investigation. A holistic approach should be followed, considering advances in key applied research aspects of the industrial application of NTTs as tools to increase the content of health-promoting compounds in whole fruits and vegetables. Figure 2 summarizes the holistic approach proposed herein to determine the feasibility of NTTs as tools to improve the content of bioactive compounds in whole fruits and vegetables.

When selecting the fruit or vegetable to investigate, it is essential to consider if it is climacteric or non-climacteric. As observed in the cited research (Table 1, Table 2 and Table 3), NTT effects on whole fruits and vegetables are tissue-dependent. For instance, in the case of climacteric crops, postharvest ripening could be decreased depending on the processing condition selected. Therefore, it is important to evaluate the physiological attributes of the fruit or vegetable to increase the understanding of the response of the tissue at the physiological level. The selected NTT processing conditions should be mild, in order to induce a stress response in the plant cell while maintaining its viability. Conditions proposed for evaluation are highlighted in Figure 2. The effect of NTT treatment on bioactive plant components, quality, and the physiological attributes of the whole fresh produce under investigation should be evaluated both immediately after processing and during storage. Evaluating the immediate effect of NTT processing determines the most intense processing conditions that can be applied in the tissue while retaining essential quality attributes and cell viability.

Once the whole tissue is treated with the NTT under investigation, the fruit or vegetable should be stored under adequate conditions used for their commercialization in order to determine the effect of the applied conditions on the biosynthesis of bioactive compounds and on the physiological and quality attributes during storage of the crop, which allow for determining its shelf-life. Physiological attributes proposed for evaluation immediately after processing and during storage include respiration rate, ethylene production, oxidative stress, cell viability, expression of secondary metabolite biosynthesis genes, and enzyme activity related to quality and biosynthesis of secondary metabolites. Likewise, quality parameters suggested for evaluation in the processed whole tissue include sensory acceptability, color, texture, soluble solids, pH, and microbial counts; in the specific case of seed treated with NTTs as pretreatments for germination, measurement of the content of natural antinutrients in the sprouts is also suggested.

Based on the results obtained regarding the effects of NTTs on secondary metabolite content and on physiological and quality attributes evaluated immediately after processing and during storage, the technological feasibility of using an NTT on the selected crop can then be determined. The economic feasibility of bioprocessing should be carefully considered when proposing the commercialization of whole fruits and vegetables treated with NTTs. The holistic approach to evaluating the technical and industrial feasibility of NTT as abiotic elicitors presented herein will allow the advancement of this research, taking these functional whole fruits and vegetables from the laboratories to the market.

## 6. Conclusions

Herein, an update regarding recent research reporting the use of NTTs as abiotic elicitors to induce the biosynthesis of health-promoting compounds in whole fruits and vegetables has been presented. Results from the reviewed literature demonstrate that NTT application under certain conditions in different crops results in the development of next-generation whole fruits and vegetables with improved bioactive compound contents and enhanced shelf-life. Moreover, the analysis presented regarding the industrial implementation and economic feasibility of NTT application in obtaining functional whole fruits and vegetables is positive, providing motivation for the scientific community to continue researching this fascinating emerging research field.

Although this paper has focused on potential industrial implementation of NTT for enhancing the health-promoting properties of whole fruits and vegetables, it may also be interesting to explore scenarios in which consumers are the ones applying NTT in their homes. Would it be possible to have affordable, practical, and safe-to-use countertop NTT equipment for residential use, allowing home processing of whole fruits and vegetables in order to increase their nutraceutical value? Currently, commercial US equipment for residential use (not necessarily designed for food applications) may partially meet the processing conditions at which abiotic stress is elicited in whole fruits and vegetables. Would it be possible to have residential PEF or HHP equipment adapted to the typical home food preparation process? This “Do-It-Yourself (DIY)” approach to NTT processing should be further explored in order to assess its technical and economic feasibility.

## Figures and Tables

**Table 3:** Effects of moderate intensity pulsed electric fields (MIPEF) on the biosynthesis of health-promoting compounds and on the quality and physiological attributes of whole fruits and vegetables.

Horticultural Crop	MIPEF Processing and Storage Conditions Evaluated	Main Findings		References
		Effects on the Biosynthesis of Health-Promoting Compounds	Effects on Quality and Physiological Attributes	
Apple *(Malus* *domestica*, var. Golden delicious)	Whole apple fruits were treated at 0.4–2 kV cm^−1^ using 5–35 monopolar pulses of 4 μs at a frequency of 0.1 Hz (energy input of 0.008–1.3 kJ kg^−1^). Samples were stored at 4 and 22 °C for 48 h.	MIPEF treatment (0.008 kJ kg^−1^) induced an increase in total phenolic (13%) and flavan-3-ol (92%) contents in fruits stored at 22 °C for 24 h, and in flavonoids (58%) in samples stored at 4 °C for 24 h.	The authors did not report quality characteristics and physiological measurements of the samples.	[38]
Tomato (*Lycopersicon esculentum* Mill. cv. Daniella)	Tomato fruits were subjected to different electric field strengths (from 0.4 to 2.0 kV cm^−1^) and number of pulses (from 5 to 30). Samples were stored at 4 °C for 24 h.	Except for 2 kV/cm treatment, MIPEF-treated tomatoes showed higher phenolic content after 24 h of treatment than the control. The increases in phenolic content of tomatoes ranged from 6.6% (five pulses at 0.4 kV cm^−1^) to 44.6% (30 pulses at 1.2 kV cm^−1^). Accumulation of lycopene was detected after storage of MIPEF-treated tomato fruit, which ranged from 0.6% (18 pulses at 2 kV cm^−1^) to 31.8% (five pulses at 1.2 kV cm^−1^) as compared with non-treated tomato.	The authors did not report quality characteristics and physiological measurements of the samples.	[39]
		MIPEF treatment at 1.2 kV cm^−1^ and 30 pulses resulted in the highest accumulation of caffeic acid-*O*-glucoside (170%), caffeic acid (140%), and chlorogenic acid (152%). MIPEF treatments at 1.2 kV cm^−1^ and five pulses presented the highest accumulation of α-carotene (93%), 9-*cis*-lycopene (94%) and 13-*cis*-lycopene (140%), respectively.	The authors did not report quality characteristics and physiological measurements of the samples.	[40]
Tomato (*Licopersicon esculentum* Mill. cv. Raf)	Tomato fruits were subjected to different electric field strengths (40, 120, and 200 kV m^−1^) and number of pulses (5, 18 and 30 pulses). Specific energy input ranged from 0.02 to 2.31 kJ kg^−1^. Samples were stored at 4 °C for 24 h.	The highest increase in carotenoid content (50%) was achieved in tomato fruits treated with an energy input of 2.31 kJ kg^−1^ (200 kV m^−1^−30 pulses).	MIPEF-treated tomatoes showed a significant increase in the respiration rate of tomato fruit. Ethylene concentration was higher (53%) in fruits subjected to the lowest electric field strength. Further increase in PEF intensity inhibited ethylene production. Acetaldehyde synthesis was induced when tomatoes were treated at energy inputs higher than 0.38 kJ kg^−1^. The higher the treatment intensity, the greater the softening effect. Total soluble solids and pH values increased with the treatment intensity.	[41]
Carrot (*Daucus carota* cv. Nantes)	Whole carrots were subjected to different electric field strengths (0.8, 2, and 3.5 kV cm^−1^) and number of pulses (5, 12, and 30). Samples were stored for 48 h at 4 °C.	The largest increase in phenolic content was observed at 24 h, after applying five pulses of 3.5 kV cm^−1^ (39.5%) and 30 pulses of 0.8 kV cm^−1^ (40.1%).	A correlation between the specific energy input and cell viability was found. After applying 3.5 kV cm^−1^, viability decreased by 87.5–79.4%. At 24 h, whole carrots treated with five pulses of 3.5 kV cm^−1^ and 30 pulses of 0.8 kV cm^−1^ showed texture softening while preserved the color.	[42]
	Whole carrots were processed in a PEF batch system. Samples were treated with five pulses of 350 kV m^−1^ (580 ± 80 J kg^−1^). Samples were stored for 36 h at 4 °C.	Immediately after PEF treatment, whole carrots showed a decrease in *p*-coumaric acid (−42.3%), protocatechuic acid (−78.1%), and ferulic acid (−56.3%). The maximum accumulation of phenolics in whole carrots was reached after 24 h of PEF treatment, where whole carrots presented a significant increase in total phenolics (80.2%) and chlorogenic (74.9%), ferulic (52.2%), and *p*-OH-benzoic (94.7%) acid compared to the control. At 36 h of storage a decrease in phenolic content was observed.	PEF induced an immediate increase in respiration. From 12 to 36 h, PEF-treated carrots presented a 123–164% higher respiration rate than untreated carrots. PEF did not induce an immediate increase in volatile organic compound production in whole carrots. However, at 12 h of storage, samples treated with PEF generated higher amounts of acetaldehyde (7 pg kg^−1^ s^−1^), ethanol (68 ng kg^−1^ s^−1^), and ethylene (50 ng kg^−1^ s^−1^), whereas these volatiles were not detected in untreated carrots. PEF application delayed the peak of maximum peroxidase enzymatic activity for 12 h. Pectin methylesterase (PME) activity increased during the first 12 h of storage in the control. In contrast, PEF induced an immediate increase (164%) in enzyme activity, which remained stable for the following storage time. Polygalacturonase (PG) activity immediately decreased by 31%–32% after treatment. Phenylalanine ammonia-lyase (PAL) activity in untreated carrots remained stable during storage, whereas PEF-treated samples showed a constant increase in PAL activity during storage, showing the highest increase (153%) at the end of the study.	[43]

**Table 4:** Comparison between non-thermal technologies (high hydrostatic pressure, pulsed electric fields, and ultrasound) in terms of their scaling-up feasibility and cost of operation/maintenance.

Non-Thermal Technology	General Principle of Action	Scaling-Up Feasibility	Cost of Operation/Maintenance
**High Hydrostatic Pressure** (**HHP**)	A high-pressure pump injects liquid into a treatment chamber where the product of interest is located. High pressure generates structural changes in tissues, cells, and molecules depending on process conditions.	**High** There is industrial equipment that can process tens of tons per hour.	**High** Due to the mechanical complexity of the equipment and the constant wearing of sealing components, the cost of maintenance is relatively high.
**Pulsed Electric Fields** (**PEF**)	Electrodes generate repetitive short electric pulses in a treatment chamber designed for solids, suspensions, or liquids. The pulsed electric field generates changes in the permeability of the cell wall and cell membrane, causing changes in metabolic flux and cell viability depending on process conditions.	**Medium** There is equipment available at industrial scales, although processing capability is somewhat limited compared to HHP.	**Medium** The equipment is robust in general terms, and the configuration of the electrodes may enhance energy efficiency.
**Ultrasound** (**US**)	Ultrasound waves are generated, propagating through a transmitting medium. As the sound wave propagates, the fluctuations in pressure generate cavitation (formation and collapse of microbubbles), promoting shear stress that affects the integrity of cells and molecules depending on process conditions.	**Low-Medium** Most of the equipment is still at pilot-plant scale. Although there are efforts for application at larger scales, the reach of sound wave propagation seems to be a limiting factor.	**Medium** The equipment is robust in general terms. The sound source elements erode constantly and must be periodically replaced. A fraction of the energy dissipates as heat, decreasing efficiency.

## Data Availability

Not applicable.
